# An Exploration of Self-Efficacy and Its Associated Factors among Rheumatoid Arthritis Patients in Taiwan

**DOI:** 10.3390/medicina60101653

**Published:** 2024-10-09

**Authors:** I-Yu Hsiao, Hanoch Livneh, Wei-Jen Chen, Ming-Chi Lu, Tzung-Yi Tsai

**Affiliations:** 1 Department of Nursing, Dalin Tzu Chi Hospital, Buddhist Tzu Chi Medical Foundation, Chiayi 62247, Taiwan; df383346@tzuchi.com.tw; 2 Department of Psychology, National Chung Cheng University, Chiayi 621301, Taiwan; 3 Division of Allergy, Immunology and Rheumatology, Dalin Tzu Chi Hospital, Buddhist Tzu Chi Medical Foundation, Chiayi 62247, Taiwan; 4 Rehabilitation Counseling Program, Portland State University, Portland, OR 97207-0751, USA; livnehh@pdx.edu; 5 Department of Chinese Medicine, Dalin Tzuchi Hospital, Buddhist Tzu Chi Medical Foundation, Chiayi 62247, Taiwan; tough2915@hotmail.com; 6 Graduate Institute of Sports Science, National Taiwan Sport University, Taoyuan 333325, Taiwan; 7 School of Post-Baccalaureate Chinese Medicine, Tzu Chi University, Hualien 97004, Taiwan; 8 Center of Sports Medicine, Dalin Tzuchi Hospital, Buddhist Tzu Chi Medical Foundation, Chiayi 62247, Taiwan; 9 School of Medicine, Tzu Chi University, Hualien 97004, Taiwan; 10 Department of Environmental and Occupational Health, College of Medicine, National Cheng Kung University, Tainan 70428, Taiwan; 11 Department of Medical Research, Dalin Tzu Chi Hospital, Buddhist Tzu Chi Medical Foundation, Chiayi 62247, Taiwan

**Keywords:** self-efficacy, rheumatoid arthritis, arthritis self-efficacy scale, factors, Taiwan

## Abstract

Self-efficacy is an important ingredient in successful disease management, especially in patients with chronic conditions such as rheumatoid arthritis (RA). However, the information on self-efficacy and its influencing factors among RA patients is scarce. This study investigated the level of self-efficacy and its pertinent predictors among RA patients in Taiwan. This cross-sectional study recruited patients with RA from a hospital in Taiwan between January and October 2023. A structured questionnaire was used to collect data on respondents’ demographic and job characteristics and included a Chinese version of the Arthritis Self-Efficacy Scale (ASES). Multiple linear stepwise regression analysis was employed to identify predictors of self-efficacy. A total of 284 RA patients were enrolled during the study period. The mean ASES score among enrollees was 1607.1, indicating a moderate level of self-efficacy (score range of 200–2000). The regression model displayed that those with higher disease activity scores, Taiwanese Depression Questionnaire scores, fatigue level, shorter disease duration, swollen upper limb joints, and no regular exercise regimen reported lower ASES scores, accounted for 46% of the total variance. The study findings may be useful for healthcare providers in identifying RA patients with low self-efficacy attitudes, a trait that appears to be linked to several medical indicators, and thus facilitating the provision of future tailored healthcare regimens.

## 1. Introduction

Rheumatoid arthritis (RA) is a chronic immune-mediated systemic disease that causes persistent joint inflammation. Because RA mainly affects persons of working age, this condition takes a heavy toll on physical functioning and work capacity [1]. Approximately 30% of the affected individuals become disabled within the first 3 years of RA onset, posing a high socioeconomic burden [2]. A recent study reports that the all-cause societal cost of RA annually is approximately USD 40 billion, and the per-capita healthcare costs for people with RA are equal to USD 20,919, triple that of those with no RA [3].

In addition to posing a profound economic burden, RA is a precursor of a wide array of comorbid conditions caused by chronic inflammation, including cancer, kidney dysfunction, lung disease, and cardiovascular disorder [4,5,6,7]. A recent study based on real-world data reports that the life expectancy of people with RA is 3–10 years shorter than the general population [8]. Successful adjustment to living with a debilitating chronic disorder, such as RA, requires that a patient possess sufficient knowledge, skills, and a positive attitude [9]. A concept developed by Albert Bandura’s social cognitive theory, namely self-efficacy, represents an individual’s belief in his or her capacity to effectively achieve specific performance attainments [10]. Since then, this concept has been viewed as a cornerstone in the field for managing chronic disease. Accumulating evidence these days shows that the higher the level of self-efficacy, the better a patient copes with the impact of a chronic illness [11,12]. One observational study of 3266 arthritis patients reports that baseline self-efficacy level significantly correlated with a higher frequency of physical activity per week and a lower self-reported pain level [13]. Hence, the rationale behind self-efficacy-based educational interventions can substantially improve individual self-management routines, which in turn would assist patients in coping successfully with subsequent manifestations of this disease [14,15]. On top of that, the topic of self-efficacy has been discussed and viewed as a priority in the recent recommendations by the European Alliance of Associations for Rheumatology (EULAR) for the management of inflammatory arthritis, as it profoundly boosts individual treatment adherence [16]. Therefore, to enable the use of tailored strategies geared at preventing or lessening the likelihood of poor prognosis, targeted assessment of self-efficacy among people with RA is of outmost importance.

Today, the global prevalence of persons living with RA is increasing. Based on the estimation from one recent review, the annual incidence of RA is 20–50 per 100,000 persons in European countries [17]; likewise, RA affects 0.6% to 1% of the adult population in the USA, with an incidence of 44 per 100,000 US adults per year [18]. In Taiwan, the estimated number per 100,000 persons living with RA has arisen from 57.7 in 2000 to 99.6 in 2007, with an increase of 73% [19]. In such a case, the majority of existing literature in Taiwanese rheumatology cohorts still focuses on epidemiological surveys [20], nursing interventions [21], and assessment of complementary therapies, like herbals and acupuncture [22,23]. Thus far, very few studies have explored the role of self-efficacy among RA patients, thus hampering the provision of appropriate rehabilitation at an early stage of the condition. To minimize this gap, the purpose of this study was to explore the self-efficacy and its associated factors among RA patients in Taiwan, with the goal of securing findings that could serve in helping patients to cope with and manage the sequelae of RA.

## 2. Materials and Methods

### 2.1. Study Design and Subjects

This cross-sectional study was conducted at a teaching hospital in Taiwan. Participants who visited the clinic of rheumatology and immunology between January and October 2023 were enrolled. The study cohort included persons who were: (1) over 20 years of age; (2) able to effectively express their opinions using oral and written communication skills; (3) able to speak Mandarin or Taiwanese; and (4) having RA diagnosis for more than 3 months and made by the rheumatologist in the target hospital using the 2010 classification criteria established by the American College of Rheumatology and the European League Against Rheumatism (EULAR) [24,25]. The excluded criteria included the following: having a serious cognitive impairment or disconinuing the participation at any phase of this study. The sample size needed in this survey was established by Cohen’s methodology [26], where á was set to 0.05, power was set to 0.8, and the effect size was set to 0.15. In accordance with the aforesaid indices and the considerations of multivariate linear regression analysis and the number of predictors involved, a sample of at least 82 subjects was required for this investigation via G-POWER 3.1 analytical software (Heinrich Heine University, Dusseldorf, Germany).

### 2.2. Assessments

Patients’ data were collected using the Chinese version of the Arthritis Self-Efficacy Scale (ASES) and an additional questionnaire that contained information on demographic and disease features. The ASES, developed by Lorig and colleagues in the late 1980s [27], assesses and classifies pain (5 items), physical function (9 items), and other symptoms (6 items), all of which are used to determine perceived self-efficacy during the past month. using the aforesaid domains. Respondents rank each item out of the total 20 items on a ten-point scale classification, ranging from 10 (very uncertain) to 100 (very certain), and overall scores are obtained by summing the scores for all answers, with higher scores indicating higher self-efficacy [27]. As of now, the scale has been so far proven acceptably reliable and valid for measuring self-efficacy across the rheumatic disease spectrum [11].

ASES has been translated into Chinese by Tsai and colleagues among Taiwanese patients with rheumatic diseases. They examined the validity of the Chinese version of ASES by comparing results with those of the Taiwanese Depression Questionnaire (TDQ) and reported a significant negative correlation coefficient of −0.69 (*p* < 0.01) [28]. In addition, they used principal factor analysis on data obtained from 258 rheumatology patients to assess the construct validity of the Chinese version of ASES. After using the Kaiser–Meyer–Olkin index, they applied principal component analysis with orthogonal rotations to extract three factors from these 20 items based on an eigenvalue greater than 1.0 and factor loading greater than 0.4; each item loaded on the 3 domains as expected, with 59.78% of the total variance explained. As for overall internal consistency, the findings showed Cronbach’s alpha coefficients of 0.82, 0.84, and 0.89 for pain, other symptoms, and physical function, respectively [28]. Cronbach’s alpha for the Chinese version of ASES was 0.94 in the current study.

The second part of the questionnaire contained information on demographic and disease characteristics, all of which were extracted from previous studies and clinical experience [29]. The demographic data collected included sex, age, marital status, education, monthly income, living status, religion, and lifestyle factors including smoking and exercising. Those who responded “currently” or “yes/past” to smoking were classified as smokers. Regular exercisers were defined if the subjects exercised for at least 20 min thrice weekly, including running, walking, stair climbing, swimming or cycling, etc. The disease characteristics included the accompanying chronic diseases (diabetes mellitus, hypertension, heart disease, stroke, or cancer), disease activity score in 28 joints (DAS28), duration of RA, body mass index (BMI), self-rating fatigue level, and depressive symptoms as assessed by the visual analog scale (0–10 scale) and TDQ, respectively. Additionally, the prescriptions of biological disease-modifying anti-rheumatic drugs (DMARDs) covered etanercept, adalimumab, infliximab, or rituximab. Participants were asked if they had ever taken biological DMARDs for more than 3 months after RA onset. All of the aforesaid clinical characteristics were confirmed by chart review.

### 2.3. Data Gathering Procedure

To safeguard enrollees’ rights, the researchers explained the purpose of this study and its procedures. Informed consent was obtained after subjects understood and agreed to take part in this survey. During the survey administration period, the researchers were available to answer any inquiries regarding the questionnaires. Those who were unable to complete the questionnaires in the allotted time were given the questionnaires and a pre-stamped addressed envelope to take home and were asked to return them within one week. To ensure patients’ anonymity, the questionnaires were returned with no identifying information on them. Throughout the study period, subjects had the option to withdraw from the study at any time. Before commencement of this study, approval was given by the Ethics Committee and Scientific Council of the Buddhist Dalin Tzu Chi Hospital on January 2023 (No. B11201014).

### 2.4. Data Analysis

All study variables were obtained during the initial entry into this study. The data normality was evaluated using the Kolmogorov–Smirnov criteria (*p* > 0.05 for all variables) and Q-Q normality plots. The data for all variables were reasonably normally distributed, allowing for analysis using parametric methods. Baseline characteristics are reported as the mean ± standard deviation (SD) for continuous variables and frequencies and percentages for categorical variables. Thereafter, the independent *t* test, analysis of variance (ANOVA), and Pearson correlation were used to analyze the bivariate relationships between baseline data and self-efficacy level. When applying ANOVA, a subsequent post hoc test with the Bonferroni procedure was performed for multiple comparisons. Variables that correlated significantly with the criterion measure (ASES scores) were entered into multiple linear stepwise regression analysis to determine significant predictors of self-efficacy. Meanwhile, to identify possible multicollinearity across self-efficacy predictive variables, we conducted collinearity analysis with the variance inflation factor (VIF) before conducting multiple regression analysis. Assumptions of normality, linearity, and homoscedasticity were tested as well. All analyses were conducted using SAS version 9.3 (SAS Institute Inc., Cary, NC, USA), and a *p*-value below 0.05 was considered statistically significant.

## 3. Results

### 3.1. Demographic Data and Disease Characteristics

Of the eligible subjects who were initially recruited as participants, a total of 284 questionnaires were returned within the study period. The mean age for the recruited patients was 54.1 years (SD = 15.4). The majority were females (78.9%), married (80.3%), had a lower educational level (below 9th grade) (54.2%), and were cohabitating (87.3%). More than two-thirds of the enrollees had a monthly income of ≤30,000 New Taiwan dollars (NTD) (70.1%) and reported having other concurrent comorbidities (60.9%). Slightly less than half reported regular exercising routines (48.2%), and approximately 80% were non-smokers. The mean TDQ in this group was 15.3 (SD = 9.1). The mean duration of RA was 9.7 years (SD, 5.6), and the average BMI, fatigue, and DAS28 were 24.1 (SD = 4.5), 3.3 (SD = 2.1), and 3.5 (SD = 1.3), respectively (Table 1).

### 3.2. ASES Subscale Scores

The mean ASES score was 1607.1 (SD = 337.56). Likewise, the subscales of ASES are shown in Table 2 as well. We noted that “physical function subscale” had the highest standardized score, followed by “other symptoms subscale” and “pain subscale”, with scores of 89.0, 79.6, and 65.6, respectively.

### 3.3. Correlations among Demographic Data, Disease Characteristics, and ASES Scores

Regarding the correlation analysis results, we noted that those engaging in regular exercise had higher self-efficacy (t, −3.45; *p* < 0.001) (Table 3). Most of the measured disease characteristics correlated with the ASES score, with the exception of comorbid conditions and the use of biological agents. For example, disease duration (*r*, −0.43; *p* < 0.001), swollen joint site (F, 38.34; *p* <0.001), DAS28 (*r*, −0.45; *p* < 0.001), fatigue (*r*, −0.35; *p* < 0.001), and TDQ score (*r*, −0.42; *p* < 0.001) all correlated negatively with ASES scores (Table 4).

**Figure 1:**
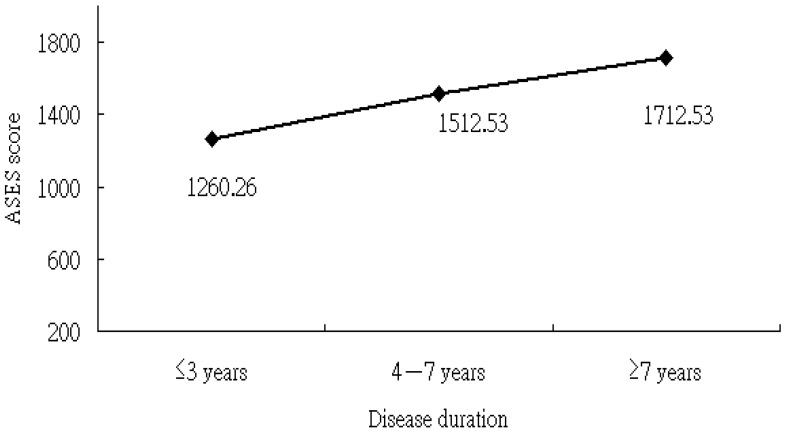
ASES score vs. disease duration of having RA. ASES: Arthritis Self-Efficacy Scale; RA: rheumatoid arthritis.

We observed a positive correlation between RA duration and the ASES score, suggesting that those with a longer disease duration since RA onset reported greater self-efficacy. We further analyzed this correlation using disease duration stratified into three time periods: ≤3 years, 4–7 years, and >7 years. The mean ASES scores for the three-time frames were 1260.26 (SD = 358.47), 1512.53 (SD = 412.66), and 1712.53 (SD = 233.79), respectively (test for trend, *p* < 0.05) (Figure 1).

### 3.4. Determination of Factors Contributing to Self-Efficacy Using Multiple Linear Stepwise Regression

After fitting multiple linear stepwise regression analysis, we identified six variables that correlated with the ASES score among RA enrollees: DAS28 score, TDQ score, fatigue level, disease duration, swollen joint site, and regular exercise or not, all of which jointly accounted for 46% of the ASES score variance (Table 4). RA patients with higher scores for DAS28, TDQ, and fatigue and those with a shorter disease duration exhibited lower self-efficacy. In addition, those who did not engage in regular exercise and those with swollen upper limbs tended to report a lower level of self-efficacy.

## 4. Discussion

Robust self-efficacy in patients may improve clinical prognoses for RA persons [12]. It is believed that lower levels of self-efficacy may hamper any advances of modern disease-modifying treatments [15]. Thus, the importance of timely assessment of self-efficacy among RA patients cannot be over-emphasized. To date, only a few studies have explored self-efficacy and its contributing factors among RA patients. A key finding from this study showed that the predictors of RA patients’ self-efficacy included those with swollen upper limb joints, higher levels of fatigue, TDQ and DAS28 scores, engaging in limited exercise regimens, and shorter disease duration, all of which explained 46% of ASES variance. These findings can help health professionals learn more about these patients. Additionally, it may serve as a reference for developing individualized strategies to help RA persons cope with the accompanying clinical manifestations.

We observed in this study that RA subjects with swollen upper limbs reported lower self-efficacy. Previous studies have not investigated ASES scores with respect to specific sites of swollen joints, making direct comparison to previous results difficult. Nevertheless, our results corroborate the findings of Walker–Bone, who showed that upper limb impairments were common among those coping with musculoskeletal disorders [30]. We speculate that RA patients with swollen upper limbs were more likely to experience difficulty in carrying out activities of daily living, such as feeding, bathing, dressing, and answering the phone, thereby decreasing their perceived self-efficacy [31]. In addition to conventional anti-arthritic treatments, rehabilitation interventions have shown the potential to restore functional independence to RA patients [32]. For example, hydrotherapy, a *water*-*based* therapeutic regimen, has been shown to substantially reduce pain and improve wrist range of motion (*p* < 0.01) [33]. Therefore, adding individually tailored rehabilitation regimens to routine care for RA patients should be considered, especially for those who grapple with severe upper limb swelling.

In addition, we noted that symptoms of depression and fatigue also correlated with lower ASES scores. This result appears to echo the concept of metacognitive therapy, which addresses the emergence of interoceptive experience of dishomeostasis throughout the body, also affecting brain functioning through immersion in distressing thoughts and feelings, which consequently act to aggravate disorder-specific negative aspects [34,35], thus abating self-efficacy motivation. Of particular importance is that these negative psychological factors often co-occur with RA [20,36]. Notably, one former study further indicated that depression may mediate the association of self-efficacy with the self-care behaviors among diabetic patients [37]. Accordingly, prior to instituting interventions to improve self-efficacy, it is recommended to have patients referred to the mental health professional for screening of psychological difficulties, including symptoms of depression and fatigue.

Findings from the present study also indicate that individuals who do not exercise regularly may experience lower self-efficacy. A similar association between exercise and self-efficacy level was reported in previous studies of people with and without associated medical conditions [38,39]. We suggest several potential reasons why exercising regularly could be associated with self-efficacy, as observed in our patient cohort. First, accumulating research shows that exercise promotes the release of endorphins, neuropeptides that aid in coping with psychological stress and cognitive function [40], which in turn enhance self-efficacy to some extent. Second, participation in group exercise classes or active hobbies with friends may provide subjects with additional benefits, such as allowing them to socialize and share meaningful moments during the exercise period, thereby encouraging them to seek out further activities that boost self-efficacy.

In our study cohort, we observed that the higher DAS28 scores markedly pertained to the ASES score; this result is in agreement with previous research findings [41,42]. In clinical practice, DAS28 is a widely adopted indicator for disease activity in RA groups, where higher values are indicative of higher disease activity. In this scenario, RA persons with higher DAS28 may experience increased peripheral joint involvement that could hamper their daily activities and, therefore, heighten their doubts about self-capability. Additionally, the duration of RA correlated positively with ASES scores. This finding is consistent with that of a previous study of self-efficacy in patients with other chronic diseases [43]. We infer that RA patients with longer disease duration have gradually learned to accept the consequences and future implications of their disability, and this may have led to higher self-efficacy. Notably, patients in our study whose RA duration was ≤3 years reported the lowest level of ASES. For this reason, healthcare providers should pay attention to newly diagnosed RA patients through the establishment of a multidisciplinary care program for evaluating patients’ psychophysical status to facilitate early referral for further therapeutic interventions.

While our study is the first to investigate self-efficacy among RA patients, the implications of the results must be considered in light of the following limitations: First, the current analysis was based on a cohort of patients at a single hospital. Although it is not unusual for study conclusions to be drawn from arbitrarily selected cohorts, a potential non-representative cohort might have introduced bias that limits the generalizability of findings to other populations and settings. Similar studies should be conducted with diverse cohorts to determine whether our findings are replicated with different demographic and geographic groups of patients with rheumatologic disorders. Nevertheless, we deliberately determined the appropriate sample size based on the research objectives, the nature of the study, the statistical power required, and relevant factors considered. Second, the cross-sectional nature of this trial might hamper further interpretation regarding the causal directionality of the identified relationships. Third, other potential confounders such as smoking habits, laboratory tests, pain level, personality characteristics and traits, and family-related factors were not assessed and thus not controlled in this study. Further research confronting these issues is warranted. Fourth, the stability of ASES scores over time could be another informative topic of study that was not investigated here.

## 5. Conclusions

Self-efficacy is an important contributor to the healthy lives of RA persons, which may reinforce their motivation to engage in health promotion behaviors more frequently. Herein, we found a trend toward lower ASES scores among RA patients with higher DAS28 and TDQ scores, fatigue, shorter disease duration, swollen upper limb joints, and a lack of regular exercise regimen. As far as clinical practice is concerned, the healthcare providers may utilize the present results to clarify the groups of patients who potentially report low self-efficacy, especially among those with newly diagnosed RA for less than three years. Not only that, but the healthcare providers would also consider these findings as a reference for designing the program towards self-efficacy improvement for RA subjects. Ensuring that holistic healthcare is available to RA patients may be a critical step to help them better psychologically adapt to their disease and possibly as important as improving the survival rate of patients with this chronic and life-threatening disease.

## Figures and Tables

**Table 1:** Demographic data and disease characteristics (N = 284).

Variables	Mean ± SD	*n* (%)
Demographic data		
Gender		
Male		60 (21.1)
Female		224 (78.9)
Marital status		
Single		56 (19.7)
Married		228 (80.3)
Educational level		
Low (below junior college)		154 (54.2)
High (above junior college)		130 (45.8)
Monthly income		
≤NTD 30,000 *		199 (70.1)
≥NTD 30,001		85 (29.9)
Living status		
Living alone		36 (12.7)
Cohabitating		248 (87.3)
Cigarette smoking		
Yes		58 (20.4)
No		226 (79.6)
Regular exercise		
Yes		147 (51.8)
No		137 (48.2)
Age (years)	54.1 ± 15.4	
Disease characteristics		
Disease duration (years)	9.7 ± 5.6	
Swollen joint site		
Upper limbs		149 (52.5)
Lower limbs		120 (42.3)
Both		15 (5.2)
Comorbidity		
Yes		173 (60.9)
No		111 (39.1)
Biological agents		
Yes		169 (59.5)
No		115 (40.5)
BMI	24.1 ± 4.5	
DAS28	3.5 ± 1.3	
Fatigue	3.3 ± 2.1	
TDQ	15.3 ± 9.1	

BMI: body mass index; DAS28: disease activity score in 28 joints; TDQ: Taiwanese Depression Questionnaire; NTD: New Taiwan dollar; * USD 1 = 32.19 at the study period.

**Table 2:** Mean and SD of the three ASES subscale scores (N = 284).

Dimension	Mean	SD	Standardized Score ^(1)^	Rank
Physical function (90–900)	801.4	145.1	89.0	1
Other symptoms (60–600)	477.4	129.4	79.6	2
Pain (50–500)	328.2	126.1	65.6	3
Total score (200–2000)	1607.1	337.6		

SD: standard deviation. ^(1)^ Standardized scores: mean ÷ total score × 100%.

**Table 3:** Comparison of ASES by participants’ baseline characteristics (N = 284).

	ASES Score			
	Mean	SD	*p*	Bonferroni
Demographic data				
Gender				
Male	1668.25	369.91	0.11 ^a^	
Female	1590.53	328.63		
Marital status				
Single	1565.35	343.31	0.31 ^a^	
Married	1617.16	337.38		
Educational level				
Low (below junior college)	1584.28	353.08	0.31 ^a^	
High (above junior college)	1617.16	319.83		
Monthly income				
≤NTD 30,000	1590.07	349.97	0.20 ^a^	
≥NTD 30,001	1646.45	308.66		
Living status				
Living alone	1533.90	297.49	0.17 ^a^	
Cohabitating	1617.55	343.39		
Cigarette smoking				
Yes	1639.44	384.16	0.54 ^a^	
No	1602.23	332.05		
Regular exercise				
Yes	1673.08	301.72	0.001 ^a^	
No	1535.99	361.99		
Age (years)			0.73 ^b^	
Disease characteristics				
Disease duration (years)			0.001 ^b^	
Swollen joint site				
① Upper limbs	1466.04	348.76	<0.001 ^c^	① > ③, ① ③ > ①
② Lower limbs	1785.36	227.34		
③ Both	1579.33	327.45		
Comorbidity				
Yes	1587.72	350.61	0.80 ^a^	
No	1637.38	318.01		
Biological agents				
Yes	1617.88	307.43	0.88 ^a^	
No	1590.21	380.19		
BMI			0.09 ^b^	
DAS28			<0.001 ^b^	
Fatigue			<0.001 ^b^	
TDQ			<0.001 ^b^	

ASES: Arthritis Self-Efficacy Scale; SD: standard deviation; BMI: body mass index; DAS28: disease activity score in 28 joints; TDQ: Taiwanese Depression Questionnaire; NTD: New Taiwan dollar. ^a^ By independent *t* test; ^b^ by Pearson correlation; ^c^ by ANOVA test.

**Table 4:** Multiple stepwise regression analysis predicting ASES scores among RA patients (N = 284).

	Standardized Coefficient *	Unstandardized Coefficient	Adjusted *R*^2^	*p*	VIF	95% Confidence Interval
DAS28 score	−0.23	−55.47	0.20	<0.001	1.14	−79.42–−31.82
Swollen joint site (Upper limbs)	−0.32	−67.97	0.30	<0.001	1.09	−99.71–−43.22
TDQ score	−0.20	−3.51	0.38	<0.001	1.19	−7.27–−0.59
Regular exercise (Yes)	0.14	86.17	0.41	0.004	1.02	32.16–112.19
Fatigue	−0.18	−23.66	0.44	0.004	1.12	−35.66–−16.69
Disease duration	0.20	88.42	0.46	0.003	1.01	67.38–107.46

DAS28: disease activity score in 28 joints; TDQ: Taiwanese Depression Questionnaire; VIF: variance inflation factor. * Positive values indicate a higher level of self-efficacy.

## Data Availability

Data are available upon reasonable request. The data involved and analyzed during the current work are available from the corresponding authors only on academic research request.
